# Smart Sensors Enable Smart Air Conditioning Control

**DOI:** 10.3390/s140611179

**Published:** 2014-06-24

**Authors:** Chin-Chi Cheng, Dasheng Lee

**Affiliations:** Department of Energy and Refrigerating Air-Conditioning Engineering, National Taipei University of Technology, Taipei 10608, Taiwan; E-Mail: newmanch@ntut.edu.tw

**Keywords:** smart sensors, mobile phone, smart air conditioner, intention causing control, energy conservation, human comfort

## Abstract

In this study, mobile phones, wearable devices, temperature and human motion detectors are integrated as smart sensors for enabling smart air conditioning control. Smart sensors obtain feedback, especially occupants' information, from mobile phones and wearable devices placed on human body. The information can be used to adjust air conditioners in advance according to humans' intentions, in so-called intention causing control. Experimental results show that the indoor temperature can be controlled accurately with errors of less than ±0.1 °C. Rapid cool down can be achieved within 2 min to the optimized indoor capacity after occupants enter a room. It's also noted that within two-hour operation the total compressor output of the smart air conditioner is 48.4% less than that of the one using On-Off control. The smart air conditioner with wearable devices could detect the human temperature and activity during sleep to determine the sleeping state and adjusting the sleeping function flexibly. The sleeping function optimized by the smart air conditioner with wearable devices could reduce the energy consumption up to 46.9% and keep the human health. The presented smart air conditioner could provide a comfortable environment and achieve the goals of energy conservation and environmental protection.

## 1. The Development of Air Conditioning Technology

For a higher quality and comfortable modern lifestyle, people rely on air conditioners (ACs) much more than before. In both developed and developing countries, ACs increase the occupancy ratio of building areas. This also leads to a rapid growth in the energy consumption by ACs. According the static data [1], HVAC almost consumed half of the energy in buildings and 20% of the overall national energy consumption. Therefore, it is important to decrease the energy consumption of ACs in residential and commercial buildings.

The methods of developing new energy-efficient equipment, applying complex control strategies, using solar energy as a new energy source, *etc.*, are all being considered for saving energy in ACs. Among them, applying control strategies may be the more economical and efficient method. Energy-efficient control strategies for controlling variable speed pumps in a central AC are illustrated by Ma and Wang [2]. The results show that the energy consumption of pumps can be lowered by using these control strategies. A feedback controller for ACs is designed and improves the energy efficiency of an AC [3]. Zhao *et al.* [4] presented a temperature- and humidity-independent control strategy to reduce the energy consumption of an AC in an office building. The experimental results show that the strategy can provide a better coefficient of performance of the AC and a comfortable indoor environment even in very hot and humid weather. For multi-unit ACs, a fuzzy logic control strategy is used to control the operational number of compressors and fans to enhance energy efficiency [5]. The previous researches of applying control strategies on ACs are based on the difference of parameters and cause the reaction. This is one kind of passive responses and may not be suitable for human comfort.

The developing history of ACs is related to the efficiency, technology, human comfort, and energy consumption. ACs' progress includes window type, split type, fixed frequency, convertible frequency, and the recently presented smart type. In the 1990s, in order to reduce noise and satisfy consumers, window type air conditioners was modified to the split type by moving the compressor outdoors. Since 2000, the high oil price and the demands for energy conservation have forced the control of compressors to be modified from fixed frequency to convertible. The fixed frequency control of compressors adopts full power as the On-Off output, and causes temperature variations and waste of energy. However, by using an electronic expansion valve, the modified convertible frequency control could adjust the compressor output and the flow rate of refrigerant continuously according to the indoor thermal mass for energy conservation.

**Figure 1.**
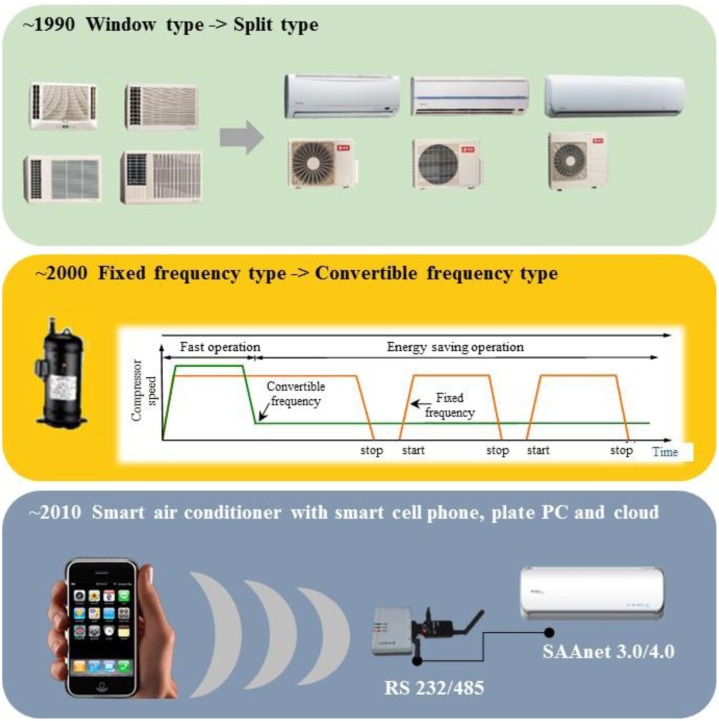
Development of air conditioning technology: (**a**) From widow air conditioner to split type, (**b**) fixed to convertible frequency, the Inverter control air conditioner (**c**) smart air conditioner.

Since 2010, mobile phones (also called smart phones), tablet personal computers, and cloud computing and 4th generation (4G) communication in 2014 were widely utilized and have caused an information revolution. These novel technologies were also adopted for the control of air conditioners, and this resulted in further air conditioning technology improvements to become smart air conditioners [6,7]. By using communication technologies, the adjustment of air conditioners is not only a single feedback of setting information. A smart air conditioner can be combined with an infrared sensor for human position sensing [8], meteorological webs for outdoor weather information, wearable devices for human activity and intention awareness. Henceforth, air conditioners are expected to adjust the indoor temperature efficiently considering human comfort and energy conservation. The development of the control of the air conditioner is briefly illustrated in Figure 1.

Figure 1 presents the progress of air conditioners from window to split type in the 1990s, as shown in Figure 1a, from fixed frequency to convertible one in the 2000s, as shown in Figure 1b, and the smart air conditioners of the 2010s, as shown in Figure 1c. In Figure 1b, the red line indicates the on-off operation of the fixed frequency type compressor, and the green line indicates the stable operation of the convertible frequency type. Figure 1c presents the newest development of the smart air conditioner enabled by mobile phone, 4G communication and the SAAnet 3.0/4.0 machine control protocol. The user may optimize the settings of an air conditioner through a mobile phone. The development tendency in Figure 1 indicates that the design criteria of an air conditioner may include human comfort and energy conservation. In this study, the mathematical modeling and development of sensors and control strategies for air conditioning are illustrated. The experimental results of fixed, convertible frequency and smart air conditioners are compared to find the solutions for the better energy conservation and human comfort.

## 2. Mathematical Modeling of Air Conditioning Control

This study focused on smart control of air conditioners using mobile phones and wearable devices as smart sensors. It's the newest trend in air conditioner development. Before introducing the design, a mathematical model of air conditioning control is derived from fundamental theorems. That would help to illustrate the following concepts clearly. The fundamental operating principle of the air conditioner is the vapor cycle introduced by thermodynamics [9]. The vapor cycle includes the following steps: (1) the saturated refrigerant is compressed to a higher pressure vapor, known as superheated vapor; (2) rejecting heat from the system by either the circulating water or air and is condensed into saturated liquid; (3) undergoing an abrupt reduction in pressure through the throttle and expansion process, and becoming a liquid and vapor refrigerant mixture with lower temperature and pressure; (4) absorbing the heat of an enclosed space by a circulating fan in the evaporator and expands into the saturated vapor; (5) vapor is routed back into the compressor to complete cooling cycle by cycle. The vapor cycle includes compression, heat extraction, expansion, and heat absorption. This process is accomplished by the compressor, condenser, expansion valve and evaporator [10]. The detailed operating principles of the vapor cycle include the variation of temperature, pressure, enthalpy and entropy, described in [10].

**Figure 2.**
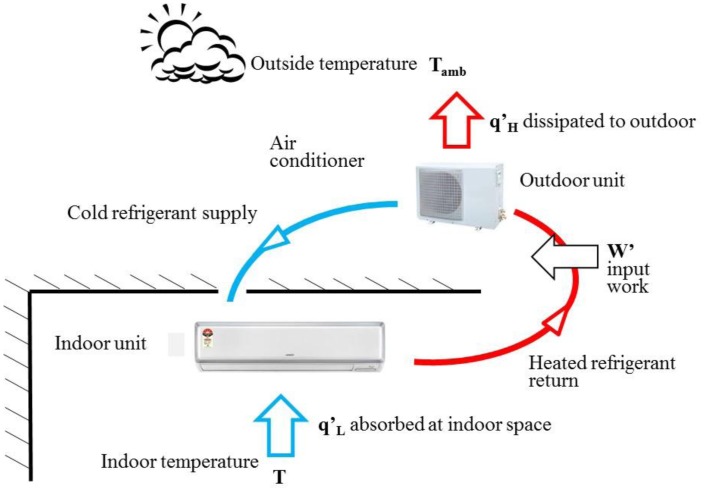
Working principals of a split type air conditioner. Smart control design will be applied to this type of air conditioner.

For a traditional window type air conditioner, these four devices were installed in an air conditioner to carry out the vapor cycle and cool down the closed space. The traditional window type air conditioner has almost been 90% replaced by the split type recently in Taiwan. The compressor and condenser of the split type air conditioner are organized in the outdoor unit to avoid the compressor noise. The expansion valve and evaporator are organized as the indoor unit. One advantage of split type air conditioner is its quietness, and this has made it become the main trend of air conditioners for home and business. Figure 2 presents the operating procedures of the split type air conditioner. In this study, smart design is only applied to split type air conditioners since they are the main air conditioning product used in Taiwan.

In Figure 2, the indoor temperature, T, is controlled by the indoor unit through the circulating air driven by the long shape cross flow fan. In this paper, the discussion of air conditioning type is based on the subtropical climate in Taiwan. The red line in Figure 2 indicates the refrigerant absorbing the heat of the indoor space, q_L_, through the evaporator and dissipating it, q_H_, to the outdoor through the outdoor unit. The blue line in Figure 2 indicates the cooled and pressure released refrigerant driven by the compressor from the outdoor unit to the indoor one for the next heat exchange process. Through the heat exchange process, the heat is absorbed from the indoor space, low temperature T, and rejected to the outdoor space, high temperature T_amb_. The heat exchange process between the indoor and outdoor units, as shown by the red and blue lines in Figure 2, would need input power w' to drive the refrigerant.

The relationship between the input power w', absorbed heat flow q_L_ and rejected heat flow q_H_ could be determined by the 1st Law (energy conservation equation) and 2nd Law (Clausius theorem) laws of thermodynamics, as shown in Equations (1) and (2):

(1) $${\text{q}}^{\prime }-{\text{w}}^{\prime }=\frac{\text{dU}}{\text{dt}},1\text{st Law}$$

(2) $$\oint \frac{\text{dq}}{\text{T}}\leq 0,2\text{nd Law}$$

where the superscript' denotes d/dt, q is the heat flow, w is the power and U is the internal energy. For air conditioning system, the internal energy is the summation of mass (M, kg), specific heat capacity (C, kJ·kg^−1^·K^−1^), and temperature (T, °C). The mass and specific heat capacity of air conditioning system are assumed as constants. Therefore, Equation (1) could be rewritten as:

(3) $$-{\text{q}}_{\text{L}}^{\prime }+{\text{w}}^{\prime }=\text{M}\cdot \text{C}\frac{\text{dT}}{\text{dt}}$$

According to Equation (3), how to reject the most heat flow q_L_ by inputting the less power w' is the main design criterion of an efficient air conditioner. In order to solve Equation (3), the governing equation of flow field, Navier-Stokes equation [11], is necessary for analyzing the temperature response in space affected by the air flow.

The circulation integral in Equation (2) includes the four air conditioning processes, *i.e.*, compression, rejection of heat, evaporation, and heat absorbtion. According to Equation (2), the air conditioner needs the input power to remove the heat flow from indoor to outdoor. Therefore, Equation (2) can be rewritten as:

(4) $$\oint \frac{\text{dq}}{\text{T}}=\frac{-\text{q}}{\text{T}}+\frac{\text{q}}{{\text{T}}_{\text{amb}}}$$

When the air conditioning space is in the steady state (the Clausius integral equals to 0), the integral result of Equation (1) is q_L_ + w = q_H_, which is the most efficient state of the air conditioner. The input power for the air conditioner is:

(5) $$\text{w}=\frac{{\text{T}}_{\text{amb}}-\text{T}}{\text{T}}\cdot {\text{q}}_{\text{L}}$$

The definition of the efficiency of air conditioner is the coefficient of performance (COP, W/W), which is the ratio of the input power w and the removed heat flow q. Therefore, Equation (5) could be rewritten as:

(6) $$\frac{{\text{q}}_{\text{L}}}{\text{w}}=\frac{\text{T}}{{\text{T}}_{\text{amb}}-\text{T}}$$

According to Equation (6), under the fixed outdoor temperature T_amb_, the higher the indoor temperature T is, the better the COP is. If the indoor temperature increases by 1 °C, the energy consumption of air conditioner could reduce by 6%.

The efficiency and energy saving inferences of air conditioner mentioned in Equations (1)–(6) are in the steady state, *i.e.*, d/dt→0. However, the practical air conditioning space is in the time variant state. Whether the air conditioning system is energy saving or not cannot be decided only by the indoor and outdoor temperature, especially when there is the controlling system to consider and the energy saving ratio is time variant.

In order to simulate the time variant situation, the governing equation of hydrodynamics, the mentioned Navier-Stokes equations [11], is necessary. The equations set, including the mass conservation, momentum conservation and energy conservation, is adopted for analyzing the temperature drop process caused by the air flow from the air conditioner in the closed space. To avoid a complex situation, the prototype of Navier-Stokes equations, Reynolds transport theorem, is utilized to analyze the energy balance process of indoor space under the air circulating environment. The Reynolds transport theorem is described as:

(7) $$\frac{\text{dB}}{\text{dt}}={∰}_{\text{CV}}\frac{\partial \text{b}}{\partial \text{t}}\rho \text{d}\forall +{∯}_{CS}\rho \text{b}\vec{\text{V}}\cdot \vec{\text{n}}\text{dA}$$

where B is the discussing goal, = B/m, m is the mass, ρ is the density of the goal, CV is the control volume, CS is the control surface, and ∀ is the volume integral, A is the surface area integral, v⃗ denotes the velocity vector and *n⃗* denotes normal vector of integral surface.

Equation (7) is utilized to analyze the variation of temperature in the air conditioning space, and discusses the effect of air conditioner. If B is the internal energy of air conditioning space, U, by combining the 1st law of thermodynamics, Equation (1) could be rewritten as:

(8) $${\text{q}}^{\prime }-{\text{w}}^{\prime }=\frac{\text{dU}}{\text{dt}}={∰}_{\text{CV}}\frac{\partial \text{u}}{\partial \text{t}}\rho \text{d}\forall +{∯}_{CS}\rho \text{u}\vec{\text{V}}\cdot \vec{\text{n}}\text{dA}$$

Where u = U/m. The variation of temperature in air conditioning space could be determined from the variation of internal energy, heat flow and input power in Equation (8). According to Equations (1–8), the human comfort (represented by the temperature variation) and the energy conservation (represented by internal energy and input power) can be evaluated for various types of air conditioners. In this study, the temperature responses in closed space and the compressor output energy with respect to the fixed frequency, convertible frequency and smart air conditioners were simulated. The analysis of the system response for the air conditioner would be practiced in the s domain, instead of the time domain. For an air conditioning space of the fixed volume, the parameters in Equation (8) could be illustrated as: indoor temperature T, outdoor temperature T_amb_, removed heat flow −q', thermal mass M (including the weight of users and the thermal mass of indoor air), total specific heat capacity C, indoor surface area A, outdoor wall thickness X and thermal conductivity coefficient k. The thermal leakage of the air conditioning space could be approximated by the temperature difference of indoor and outdoor space as:

(9) $$\text{kA}({\text{T}}_{\text{amb}}{-\text{T)}/}_{\text{x}}$$

**Figure 3.**
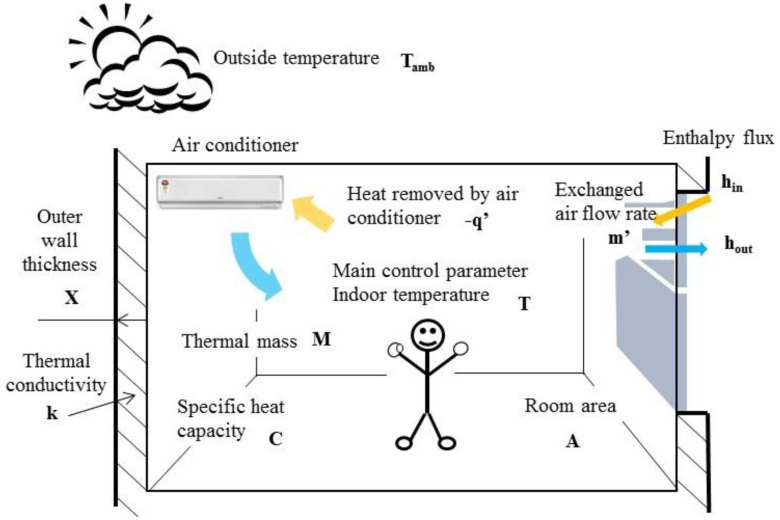
Mathematical modeling of temperature control in the air conditioning space.

To maintain the air quality of the indoor space, according to the construction technology rules of Architecture and Building Research Institute, Ministry of the Interior, the minimum mechanical ventilation is 10 m^3^/h for unit surface area m^2^ [12]. The thermal leakage due to the exchanging air flow is:

(10) $${{\text{m}}^{\prime }\text{ C}}_{\text{p}}({\text{T}}_{\text{amb}}-\text{T)}$$

where m' is the exchanging air flow, and the product of heat capacity and temperature is the enthalpy of thermodynamics. According to Equations (8)–(10), Figure 3 presents the modeling of temperature control in the air conditioning space.

Based on the model of Figure 3, Equation (8) could be rewritten as:

(11) $$-{{\text{q}}^{\prime }+}^{{\text{kA(T}}_{\text{amb}}-\text{T})}{/}_{\text{X}}=\frac{\text{d}}{\text{dt}}{∰}_{\text{CV}}\rho \text{ud}\forall {-{\text{m}}^{\prime }\text{ C}}_{\text{p}}{\text{(T}}_{\text{amb}}-\text{T})$$

Assuming T_amb_ is time-invariant, Equation (11) could be rewritten as:

(12) $$-{\text{q}}^{\prime }=\text{M}\cdot \text{C}\frac{\text{d}({\text{T}-\text{T}}_{\text{amb}})}{\text{dt}}+(m^{\prime }\text{Cp}+\frac{\text{kA}}{\text{x}})\cdot ({\text{T}-\text{T}}_{\text{amb}})$$

Taking the Laplace transform of Equation (12), the result is:

(13) $$-{\text{q}}^{\prime }\text{ (s)}=\text{M}\cdot \text{C}\cdot \text{s}\cdot \text{T(s)}+({\text{m}}^{\prime }\text{ Cp}+\frac{\text{kA}}{\text{x}})\cdot \text{T}(\text{s})$$

**Figure 4.**
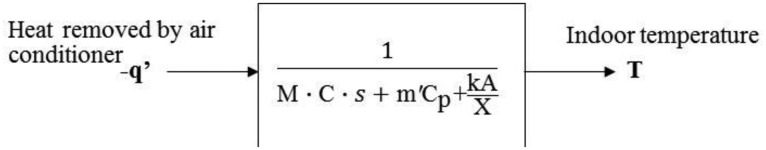
Transfer function model of air conditioning space converting from Mathematical modeling.

The transfer function model of the air conditioning space could be calculated from Equation (13) [13], as shown in Figure 4.

**Figure 5.**
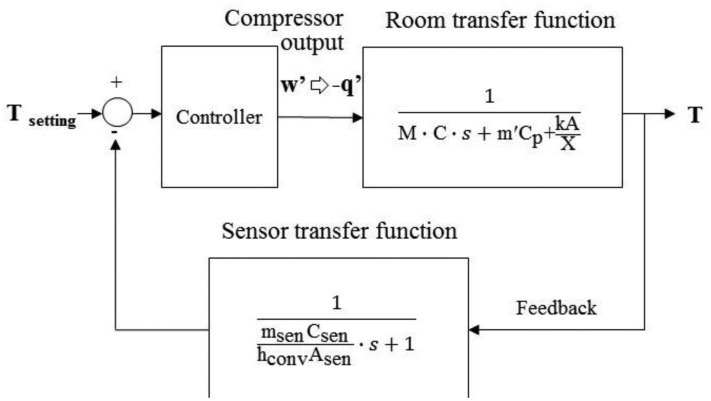
A closed-loop air conditioning space model with the feedback signal of temperature sensor.

By installing the temperature sensor in the evaporator of indoor unit of split type air conditioner for signal feedback, the open-loop air conditioning space model in Figure 4 would become a closed-loop model [14], as shown in Figure 5.

**Figure 6.**
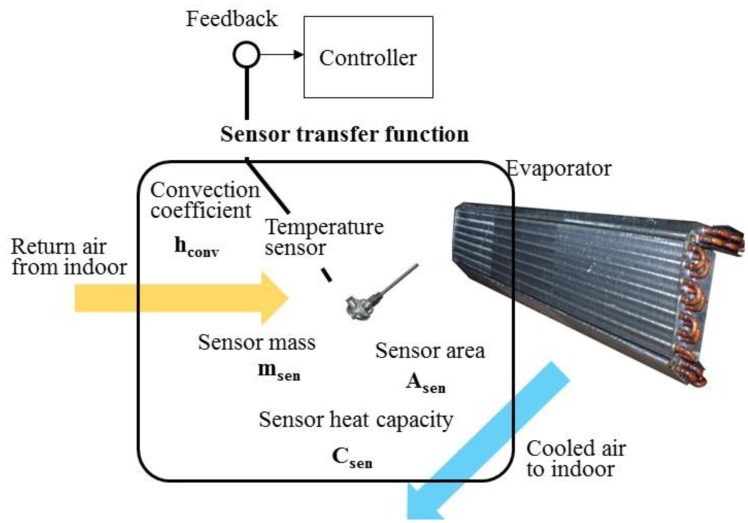
Traditional air conditioner uses a temperature sensor installed on the evaporator of indoor unit for feedback control. It yields a sensor transfer function to enable the difference causing control.

It is noted that, in Figure 5, a closed-loop air conditioning space model includes the room transfer function and sensor transfer function. The thermal mass of temperature sensor, located in the center of evaporator, would cause the convection effect of air flow and the temperature difference between the return air and sensor. The detail of the sensor transfer function is further shown in Figure 6.

According to Figure 6, the relationship between the return air temperature, T, and measured temperature by sensor, T_sen_, could be written as:

(14) $${\text{m}}_{\text{sen}}{\text{C}}_{\text{sen}}\frac{{\text{dT}}_{\text{sen}}}{\text{dt}}={\text{h}}_{\text{conv}}{\text{A}}_{\text{sen}}({\text{T}-\text{T}}_{\text{sen}})$$

Take the Laplace transform of Equation (15), and the result is:

(15) $${\text{(m}}_{\text{sen}}{\text{C}}_{\text{sen}}\cdot \text{s}+{\text{h}}_{\text{conv}}{\text{A}}_{\text{sen}})\cdot {\text{T}}_{\text{sen}}(\text{s})={\text{h}}_{\text{conv}}{\text{A}}_{\text{sen}}\cdot \text{T}(\text{s})$$

Therefore, the sensor transfer function in Figure 5 could be calculated from Equation (15) as $\frac{{\text{T}}_{\text{sen}}}{\text{T}}=\frac{1}{\frac{{\text{m}}_{\text{sen}}{\text{c}}_{\text{sen}}}{{\text{h}}_{\text{conv}}{\text{A}}_{\text{sen}}}\cdot \text{s}+1}$, as showed at the functional block diagram at the bottom of Figure 5.

The room transfer functions of air conditioning space, sensor and controller are organized to simulate the responses of indoor temperature and compressor output for air conditioning control design. As illustrated in Figure 6, a traditional air conditioner uses a temperature sensor for feedback. It causes differences causing control problems. That means the control output relies on sensing differences between setting point and real feedback signal. The difference causing control works for either fixed or convertible frequency air conditioners. By error status feedback from sensor, the compressor of fixed frequency air conditioner is switched on or off, so called On-Off control. With respect to errors, the convertible frequency one changes compressor output power to the adjust room temperature by inverter control.

As illustrated in Figure 1, these two methodologies have been transformed to smart control. By communication with a mobile phone, the novel air conditioner has the ability to get human intention feedback. For an example, the global position system (GPS) on mobile phone may sense that man will come home immediately. Then the air conditioner can directly cool down the space before sensing the temperature differences. It's thus called intention causing control. That's also the main development of smart air conditioner for reaching the goals of human comfort and energy conservation. The design will be discussed in detail in the following section.

## 3. Smart Controller Design Based on Smart Sensors

Before introducing the smart control, the On-Off and Inverter control are briefly described in the following two sub-sections. Both of them, compared with smart control, represent the difference causing control. Then the smart sensors, including mobile phone, wearable device and other sensors, are introduced. They are the key elements to determine human intentions. Finally, the design of a smart controller based on smart sensors is illustrated to illustrate the intention causing control.

### 3.1. On-Off Control of the Fixed Frequency Air Conditioner

Fixed frequency means that the compressor of the air conditioner operates in the fixed rotation speed. The relationship between the rotation speed of motor and electrical frequency can be described as:

(16) $$\text{Speed}(\text{rpm})=\frac{120\times \text{f}}{\text{p}}(1-\text{d)}$$

where f is the electrical frequency, p is the magnetic poles of motor, d is the rotary slip difference (d = 0 under zero loading). The indoor temperature is kept stable by turning the compressor on and off, when the motor operates in the fixed rotation speed and the indoor air conditioning load is less. This operating method is also mentioned as On-Off control.

**Figure 7.**
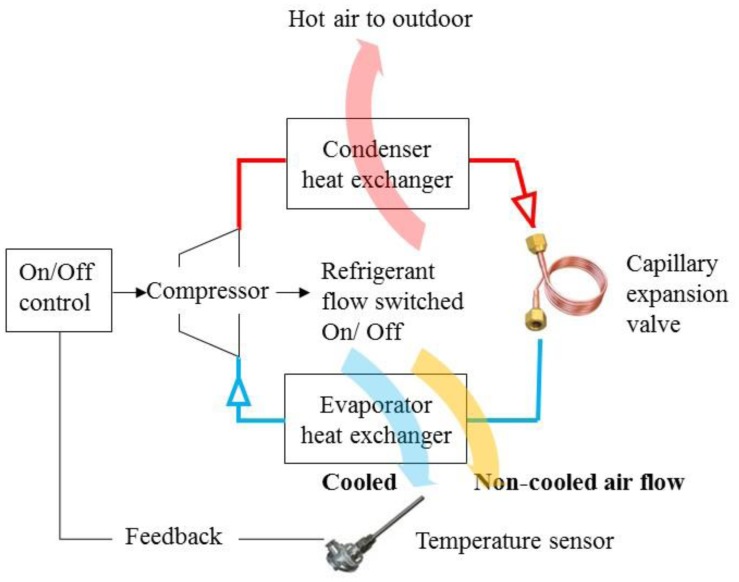
The control scheme of a fixed frequency air conditioner with On/Off control: It adopts a temperature sensor as the feedback to determine the ON/Off status of compressor. Cooled and non-cooled air flow will be delivered to air conditioning space to adjust the differences between feedback temperature and setting point. It's a kind of difference causing control.

Under the On-Off control structure, the fixed frequency air conditioner turns on the compressor when the return air temperature is higher than the set one, and turns it off on the contrary. By doing this, the refrigerant would flow through the condenser and evaporator for heat exchanging with the indoor and outdoor air to keep the indoor temperature stable. The control scheme is described in Figure 7.

### 3.2. Inverter Control of the Convertible Frequency Air Conditioner

**Figure 8.**
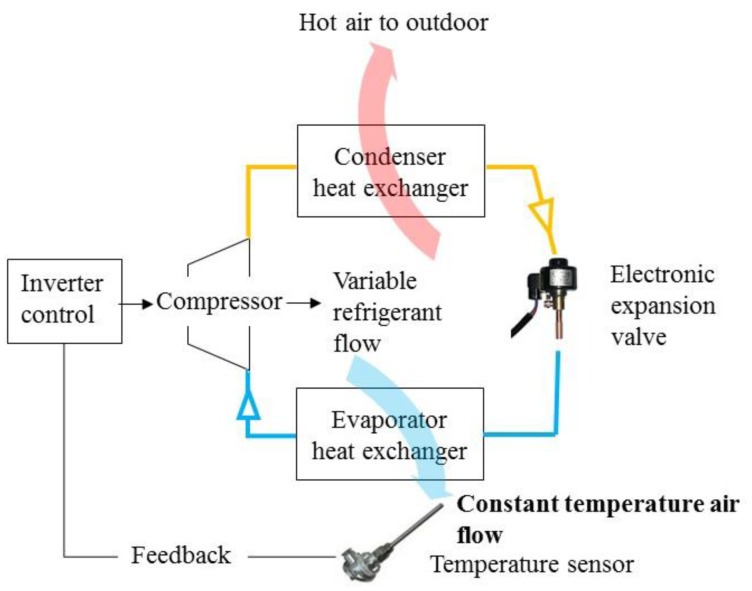
The control scheme of a convertible frequency air conditioner with Inverter control: different from the fixed frequency air conditioner, convertible frequency one has an electronic expansion valve to adjust a refrigerant flow. Inverter control changes the output continuously with respect to the different refrigerant flow rate. Even though the Inverter control would keep the constant temperature of cooling air flow, it's still a difference causing control.

The convertible frequency air conditioner is the main air conditioning product for energy conservation. It is defined as the situation where the motor rotation speed could be adjusted continuously for regulating the refrigerant energy output by changing the input electric power frequency. One can refer to Equation (16) to check how the rotation speed changes with respect to the input frequency. The control scheme is illustrated in Figure 8.

Comparing with the fixed frequency control, the convertible frequency type could first rectify the 60 Hz electricity into the direct current type, and then modulate the frequency of output electricity continuously by pulse width modulation (PWM). According to the feedback temperature difference, the air conditioner could provide the air flow of stable temperature to control the temperature of indoor space by adjusting the refrigerator flow.

The center control scheme of convertible frequency type is the “PID(s)” (proportion, integration, differentiation) function, described as:

(17) $${\text{K}}_{\text{p}}+\frac{{\text{K}}_{\text{i}}}{\text{s}}+{\text{K}}_{\text{d}}\cdot \text{s}$$

where K_p_, K_i_ and K_d_ are the constants. The PID controller could be carried out by the inverter circuit, and it is also called as the Inverter control. The benefits of Inverter control are stable air flow temperature and energy conservation. In this paper, the control stability of the temperature and the energy consumption of the fixed and convertible frequency air conditioners will be compared quantitatively.

### 3.3. Smart Sensors

**Figure 9.**
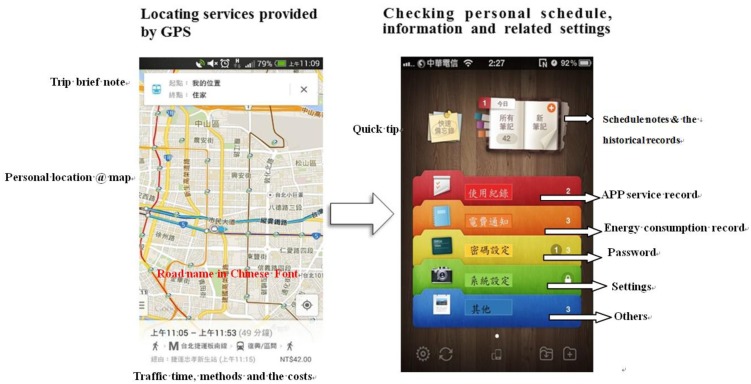
Smart sensor 1: Mobile phone providing occupants' position and intention by connecting to the GPS and personal schedule.

Smart sensors, including mobile phones, wearable devices and other sensors, are introduced. They are the key elements of smart control for obtaining the human's intention. Mobile phones would provide occupants' information by connecting to the GPS and personal schedule for collecting the position and intentions. The air conditioner could cool down the indoor temperature rapidly before the occupants enter, as shown in Figure 9.

**Figure 10.**
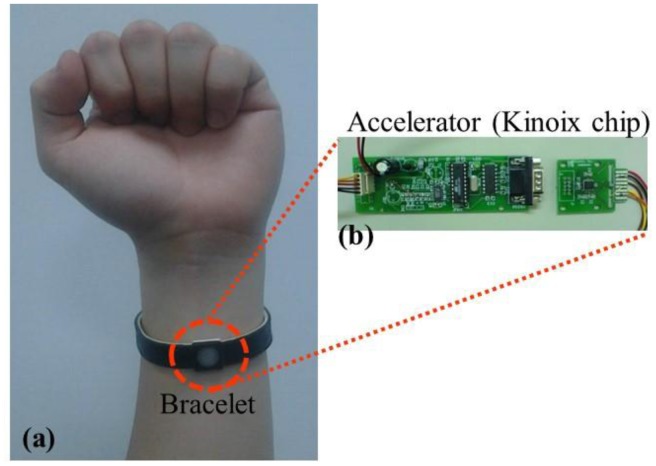
Smart sensor 2: (**a**) bracelet combining with (**b**) accelerator (Kinoix chip).

The wearable devices and their applications are predicted to have an exploding increase in the coming future [15]. Wearable devices, such as watches or bracelets, may be adopted for detecting the human sleeping state as the feedback signals of the sleeping function. Figure 10a,b present bracelet with a digital accelerator (Kionix IC type) developed in our lab. The accelerator could detect the acceleration between 10 and 10^−6^ g, the velocity between 10 and 1.67 × 10^−9^ m/s, and the displacement between 10 and 2.78 × 10^−13^ m. It can collect human motion information, and feedback to the smart air conditioner for further control.

### 3.4. Smart Control Based on Smart Sensors

Including the comparison of the fixed and convertible frequency control schemes, the quantitative analysis of the smart control based on the smart sensors is the main target in this paper. Smart control, based on the information collected by the use of mobile phones and wearable devices, intensifies the interaction with occupants and carries out the intention causing control. It may include the following aspects:

(1) Mobile phones with GPS and personal schedules, for detecting the occupants' position and intentions, could foresee the occupants' intention of entering the enclosed space. At this moment, the compressor, which is off in the general situation, could turn on in the full power. Before entering, the circulating fan turns on at the highest speed, and the air deflector swings for 10 min to enhance the air circulation. Therefore, smart control may enable the enclosed space could be cooled down rapidly after the occupant enters.
(2) The bracelet with the accelerator could detect the movement of occupants while the sleeping. After the occupant falls into a deep sleep, the air conditioner would lift the indoor temperature flexibly to avoid energy consumption.

The smart air conditioner could adjust the compressor output actively according to the occupants' active intention (going home) and passive one (falling into a deep sleep) for the goals of human comfort and energy conservation.

**Figure 11.**
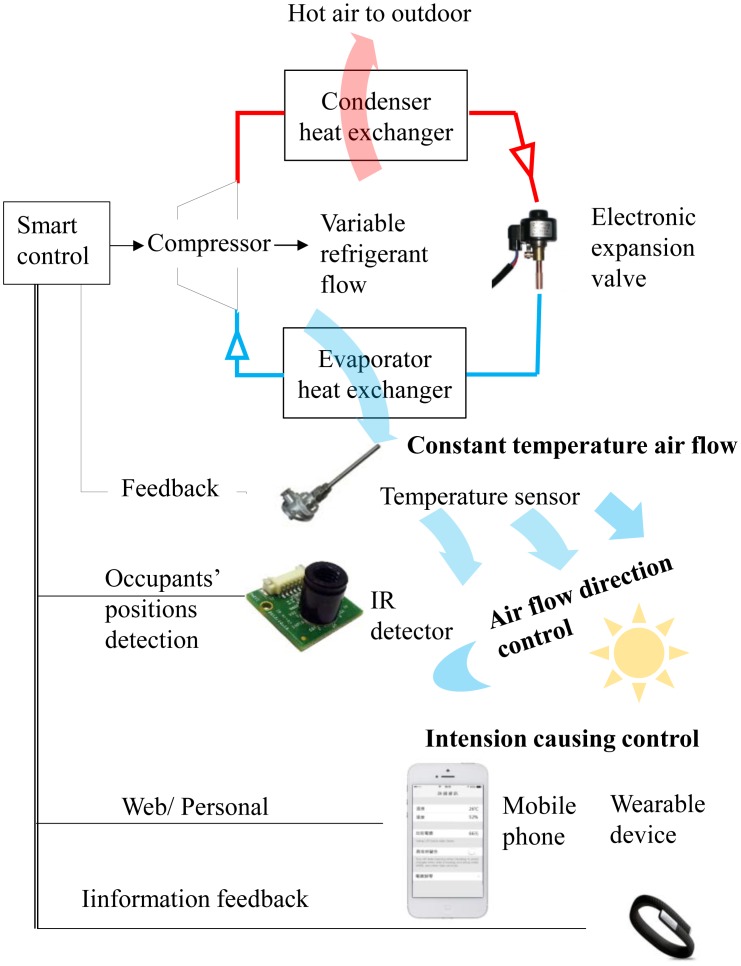
The control scheme of a smart air conditioner with Inverter control: different from the fixed and convertible frequency air conditioner with difference causing control, it's an intention causing control that adjusts the compressor output actively according to the occupants' active intention (going home) and passive one (falling in a deep sleep) for the goals of human comfort and energy conservation.

The smart air conditioner includes the following devices: (1) air conditioner with adjustable refrigeration power; (2) novel sensors capable of interacting with occupants; (3) communicating units for mobile phone and network. Figure 11 shows the controlling structure of smart air conditioner, including air conditioner, temperature sensor, IR detector, mobile phone and wearable devices.

Compared with Figures 7 and 8, the control structure of the smart air conditioner in Figure 11 utilizes a multi-sensor system to achieve smart control. The indoor infrared sensor can detect humans' position and carry out the air flow direction control. Mobile phones with GPS and personal schedules, can be used to detect the occupants' position and intention. Wearable devices can be the bracelet with the accelerator. It detects the movement of occupants while sleeping. After the occupant fallings into a deep sleep, the smart air conditioner would uplift the indoor temperature flexibly to avoid energy consumption.

**Figure 12.**
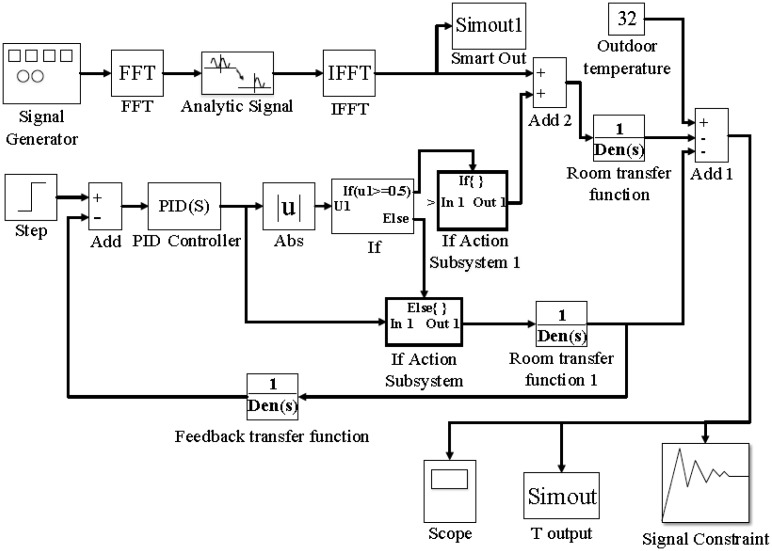
Smart control scheme based on the mathematic analysis of Section 2. It will be carried out in the electric circuit after the stability analysis of the controlling method by Matlab software.

In this paper, the smart control logic will be developed from the air conditioning principles and the controlling logics of fixed and convertible frequency air conditioners. Figure 12 presents the developed smart control scheme developed using the Matlab software. The control scheme is based on the mathematic analysis of Section 2. It will be carried out in the electric circuit after the stability analysis of the control method.

The definition of “smart control” is the control methodology, based on the multiple collected information, able to predict the demand of air conditioning space and adjust the output of air conditioner in advance. In Figure 12, the model of smart control combines the IF function of the fixed frequency On-Off control, PID function of the convertible frequency Inverter control and the developed Fast Fourier Transform (FFT) function for reacting to the demands for space air conditioning in advance and reaching the goals of human comfort and energy conservation.

The design of the smart control is different from the control logics of IF and PID functions, which is difference causing control. For the On-Off and Inverter controls, when the indoor temperature is higher than the setting value, the compressor starts to operate according to the designed controlling logics. This would result in the energy waste and human uncomfortableness. For the developed smart control, based on the multiple information from the multiple sensors, mobile phones, network and sensor network, the air conditioner is able to provide the proper response by the FFT function of the predictive control before the demand occurs. This will solve the present problems of unstable temperature and energy waste. This may be called intention causing control. This control strategy will be predominant the development of smart air conditioners in the coming future. It can foresee the refrigerant output of the air conditioner precisely. All these developments would dominate the manufacturing industry improvement and business environment of air conditioners and electricity.

## 4. Experiments

**Figure 13.**
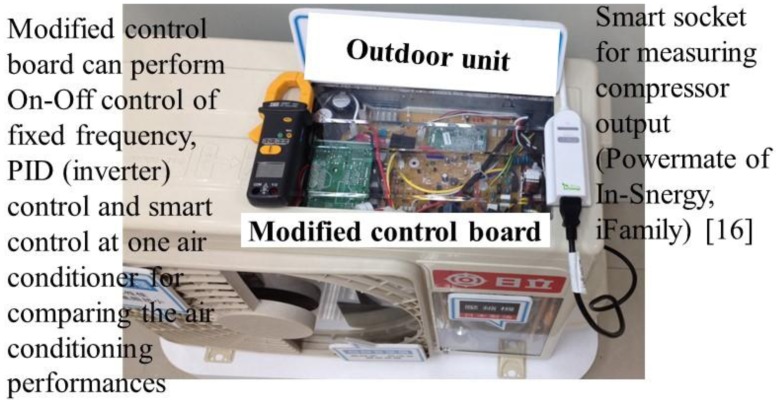
Modified controlling circuit plate at the outdoor unit of the air conditioner, and digital power meter embedded smart socket for measuring the compressor output.

**Figure 14.**
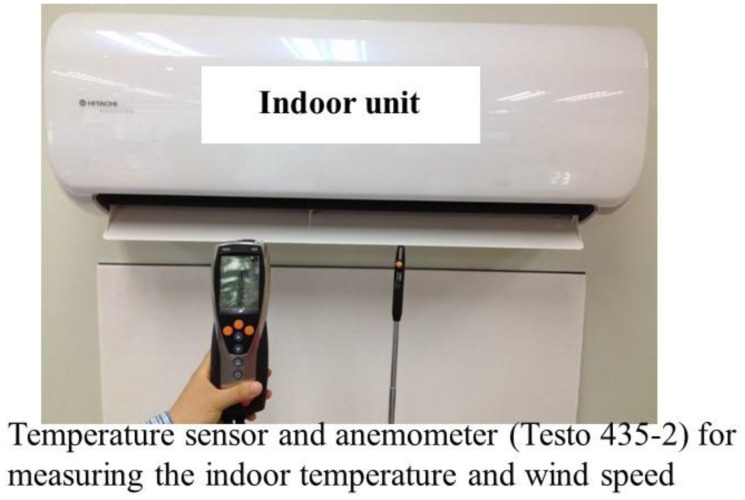
Experimental setup of temperature sensor and anemometer for measuring the indoor temperature and wind speed.

One convertible frequency air conditioner with a cell phone communication function for carrying out the smart control was chosen. After modifying the control board, the chosen air conditioner could perform the mentioned controlling strategies, *i.e.*, On-Off control of fixed frequency, Inverter (PID) control of convertible frequency, and smart control. The power consumption and cooling time of these three controlling strategies will be compared in the following sections. Figure 13 demonstrates the modified controlling circuit plate of the air conditioner, and the digital power meter (Powermate of In-Snergy, iFamily) [16] for measuring the compressor output. Figure 14 shows the experimental setup of temperature sensor and anemometer (Testo 435-2) for measuring the indoor temperature and wind speed.

In Figure 13, the compressor output is measured by the digital power meter embedded smart socket. The specification is illustrated in Table 1.

In Figure 14, the indoor temperature and wind speed could be measured by the temperature sensor and anemometer (Testo 435-2). The specification of the temperature sensor and anemometer is illustrated in Table 2.

For the air conditioners with the fixed (Type 1) and convertible frequency controls (Type 2), the temperature sensor located in the center of evaporator would send back the measured temperature as the feedback signal. This is so- called difference causing control. The smart air conditioner includes multiple feedback devices, such as IR detectors, mobile phones, and wearable device. Based on the feedback of smart sensors, the FFT function predicts the optimal setting of the air conditioner and adjusts the indoor temperature before the occupant's entrance. This is also called the intention causing control, and is the smart control tested in this study (Type 3).

There are two experimental cases for comparing these three types of air conditioners with respect to difference causing control and intention causing control. The experimental conditions for the 1st case are: the indoor space is 26.48 m^2^, three persons of average weight (70 kg) as the air conditioning load. The air conditioners with three control logics, such as fixed frequency, convertible frequency and smart control, turn on for two hours separately. The energy saving ratio is calculated from the compressor output energy. The temperature response is recorded to represent the human comfort status.

The experimental conditions for the 2nd case are: an indoor space of 13.24 m^2^, one person of 70 kg weight as the air conditioning load. The air conditioners with convertible frequency and smart controls turn on for 8 h at the night separately. The air conditioner with convertible frequency control operates in the sleeping mode. In this mode, the indoor space temperature starts at the set one and increases in the speed at 1 °C/h in the following period until reaching 28 °C. For the air conditioner with the smart control, the operating mode would include multiple information as the feedback. The information comes from the personal wearable devices. The accelerometer of the bracelet would detect human activity and determine the sleeping state for adjusting the indoor space temperature accordingly. This operating mode of smart control would reach the goals of energy conservation. The energy saving ratio is calculated from the compressor outputs of two control methodologies, sleeping mode with Inverter control and smart control. Due to the limitation of experimental environment, the testing temperature is set in the range from 21 to 32 °C.

## 5. Results and Discussion

The expected goals of smart control for air conditioner include human comfort and energy conservation. They could be evaluated by:

(1) Human comfort represented by temperature response: the time to reach the indoor temperature of the setting value, oscillating amplitude of temperature after the steady state and the level of temperature shift.
(2) Energy conservation calculated by compressor energy: a smart socket is used for measuring compressor output during two experiment cases.

**Figure 15.**
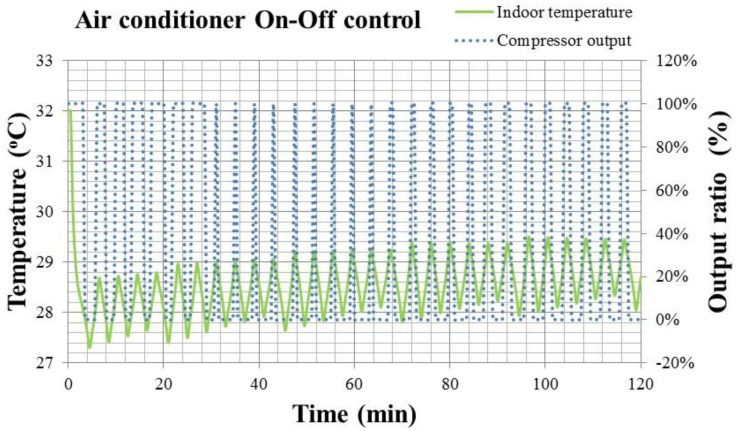
The results of indoor temperature response and compressor output for the fixed frequency air conditioner with On-Off control.

For On-Off control of the fixed frequency air conditioner, the results of indoor temperature response and compressor output from the beginning to the steady state of 28 °C are shown in Figure 15.

In Figure 15, the solid line is the indoor temperature response, and the dotted line is the compressor output. When reaching the setting value of 28 °C, the compressor will be turned off/on to adjust the refrigerant. This will result in the compressor output ratio oscillating between 0 and 100%, and the indoor temperature also oscillating between ±0.6 °C. It is also noted that the indoor temperature increases from 27.4 °C to 29 °C gradually, due to the error of the temperature sensor feedback being within ±0.2 °C. In Figure 15, the accumulated errors of temperature feedback make the switching timing of the compressor occur later, and the indoor temperature also becomes higher subsequently. Therefore, the On-Off control obviously has problems resulting from feedback errors, especially the accumulated error.

**Figure 16.**
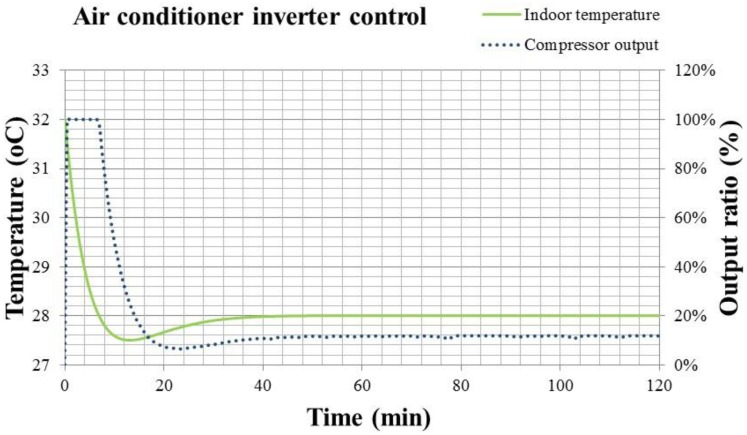
The results of indoor temperature response and compressor output for the convertible frequency air conditioner with Inverter control.

For Inverter control of convertible frequency air conditioner, the results of the indoor temperature response and the compressor output from the beginning to the steady state of 28 °C are shown in Figure 16.

**Figure 17.**
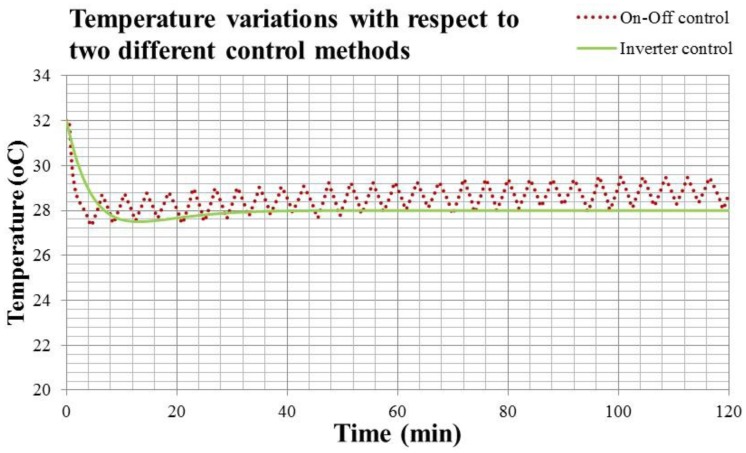
The indoor temperature responses of the Inverter and On-Off controls.

In Figure 16, the solid line is the indoor temperature response, and the dotted line is the compressor output. The compressor output decreases from 100% to a stable value of 12%. The indoor temperature keeps at 28 °C, with the error less than ±0.1 °C. Inverter control presents a more stable response of indoor temperature and compressor output than the On-Off one. However, the control output still depends on the error of temperature feedback. For the Inverter control, the PID algorithm in Equation (17) times the errors with a constant, differentiates the errors by time and accumulates the errors. All the complicated calculations take a longer time for the air conditioner to adjust the refrigerant, and the indoor temperature may reach the set value later, after 8 min. The comparison of the indoor temperature responses between the fixed and convertible frequency air conditioners is shown in Figure 17.

In Figure 17, the solid line is the indoor temperature response of On-Off control, and the dotted line is that of the Inverter control. Comparing these two responses in Figure 17, it is clear that the temperature response of the Inverter control is more stable. However, the response time of the Inverter control is slower than that of On-Off one. According to the comfort index, Predicted Mean Vote (PMV) defined by ISO-7730 [17], an indoor temperature higher than 28 °C indicates a slightly warm and uncomfortable environment. That means the occupant must endure the discomfort of the environment for around 8 min when using a convertible frequency air conditioner. Compared with the fixed frequency one, the occupant only needs to tolerate around 4 min.

**Figure 18.**
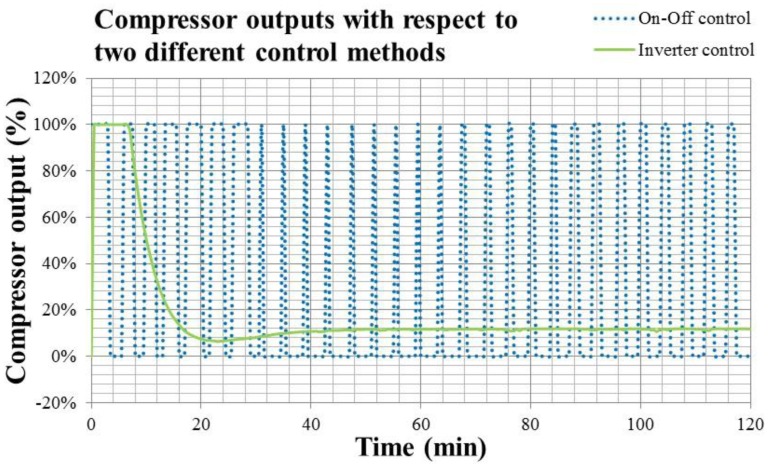
The compressor output (in % related to full load) of On-Off and Inverter control air conditioners.

The comparison of the energy consumption between the fixed and convertible frequency air conditioners can be evaluated by compressor output, as shown in Figure 18.

In Figure 18, the solid line is the compressor output of On-Off control, and the dotted line is that of the Inverter control. The compressor output is denoted by percentage related to the full power of 0.825 kW. Since this study adopts the same air conditioner to simulate the operations in fixed and convertible frequency mode, the compressor output could be viewed as the energy consumption. By integrating the total compressor outputs of On-Off and Inverter control in Figure 18, the calculated results indicate that energy consumption of a convertible frequency air conditioner is 45.4% less than that of the fixed frequency one within two-hour operation of the air conditioner.

From Figures 17 and 18, the drawbacks of the difference causing control can be observed clearly. Both On-Off and Inverter control methodologies operate according to the temperature difference between the setting one and measured one from the sensor, then adjust the compressor output in a postponed pattern. If the occupant wants to cool down the air conditioning space rapidly and adopts the On-Off control, he may suffer the temperature fluctuation. On the other hand, if the occupant won't suffer the fluctuating situation and uses the Inverter control, he may endure the discomfort for almost double the response time. When using the PID algorithm, the integration of errors causes the system response to be slow. How to achieve the rapid cool down and stable temperature control is still a problem for the existing control systems of air conditioners.

In this study, smart control may provide a solution. Smart air conditioners adopt a convertible frequency air conditioner to carry out diversified functions through the connection with smart phones. The smart phone would provide occupants' information by connecting to the GPS and personal schedule for collecting the position and intention information. The air conditioner could cool down the indoor temperature rapidly before the occupants' enter. Then, by connecting with the data of humidity, wind speed and direction, smart control is able to predict the demand of the air conditioning space and adjust the output of air conditioner in advance according to human's intentions. Therefore, this smart control is also called intention causing control.

**Figure 19.**
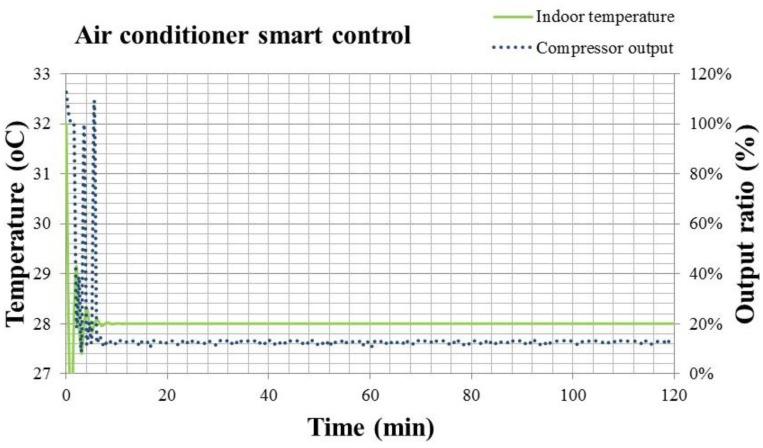
Indoor temperature response and compressor output of smart air conditioner with smart control.

For a room with 26.48 m^2^ space, occupied by three persons of average weight (70 kg) as the air conditioning load, the temperature response and the compressor output ratio according to the setting point estimated by the smart control function are shown in Figure 19.

**Figure 20.**
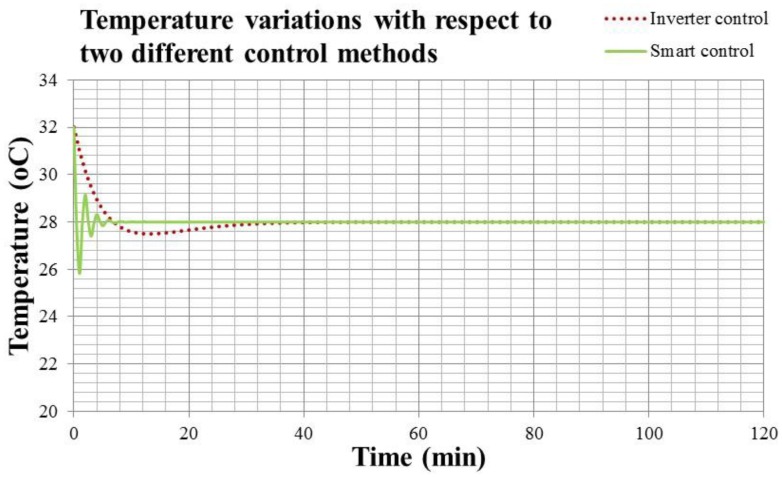
The temperature responses of smart control and Inverter control.

Figure 19 shows the temperature response and compressor output of air conditioner with smart control. It's noted that the compressor output sometimes exceeds 100%, indicating that the output over 0.825 kW can be activated sometimes under the adjustment of smart control. Due to the design tolerance, it's possible to have a transient output higher than the rating power of compressor. However, it can only be achieved by the smart control, because this control methodology speeds up the cooling fan of evaporator before turning on the compressor. All these adjustments make the evaporator of air conditioner work under good ventilation and can vaporize the refrigerant rapidly. Therefore, the compressor could provide more power to compress the refrigerant for a short period, and the cooling capacity is enhanced for cooling down the air conditioning space rapidly. Figure 20 presents the temperature responses of smart control illustrated in Figure 11 and Inverter control in Figure 8. The steady state indoor temperature is kept at 28 °C.

In Figure 20, the dotted line is the temperature response of Inverter control, and the solid line is that of the smart control. The temperature response of smart air conditioner in Figure 20 is faster than that of convertible frequency control. It is even faster than that of the fixed frequency control. Only 1.5 min is needed to reach the setting temperature. However, its stability is similar with that of the convertible frequency control. This means that the comfort level practiced by the smart air conditioner is better than that by the fixed and convertible frequency ones. Though the compressor output may exceed the rating power for a short time, the working power at the steady state is lower than the convertible frequency air conditioner with Inverter control. One can compare the compressor outputs of two control methodologies in Figures 16 and 19 to know that. The energy consumption of the smart air conditioner is 3% less than that of convertible frequency one, and 48.4% less than the fixed frequency one. In summary, the energy consumption of the smart air conditioner is 3% less than that of convertible frequency one.

Hence, the smart control could predict the air conditioning demand by collecting the occupants' information, such as position and personal schedule, through the GPS and mobile phones. This information would be analyzed by the FFT function in the frequency domain and provide the proper feedback response. That yields the “intension causing control” and solves the fundamental problem of On-Off and Inverter controls, which is “difference causing control”. Through the smart control, rapid cool down, less than 2 min, and stable temperature control with errors less than ±0.1 °C are achieved to benefit the human comfort and energy conservation.

In addition to benefit human comfort and energy conservation, the smart air conditioner is also useful when humans are sleeping at night. The experimental setup is described above. Mobile phones and wearable devices like bracelets equipped with accelerometer, are adopted for detecting the human sleeping state as the feedback signals of the sleeping function. They could collect the information of human temperature, pulse and action, and feed it back to the smart air conditioner for further control. The sleeping function of traditional air conditioner would increase the temperature in the speed of 1 °C/h until the setting value of 28 °C. However, the sleeping function of the smart air conditioner with the wearable devices would adjust the temperature setting right after the user enters deep sleep.

**Figure 21.**
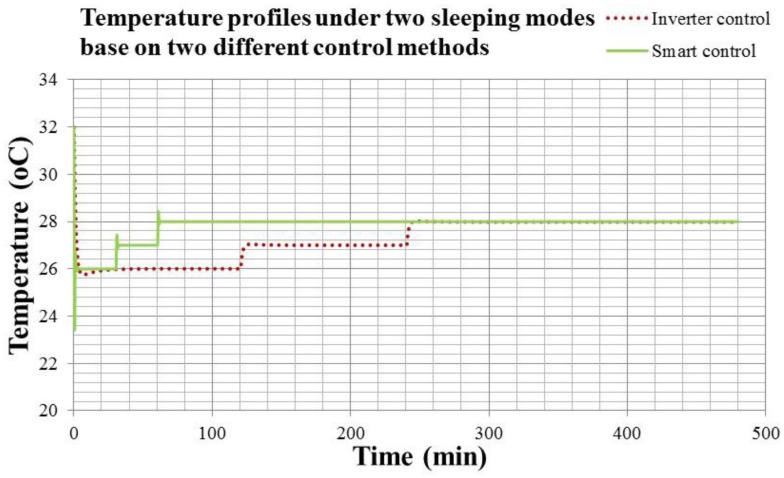
The temperature response of sleeping functions in the night under the Inverter and smart controls.

**Figure 22.**
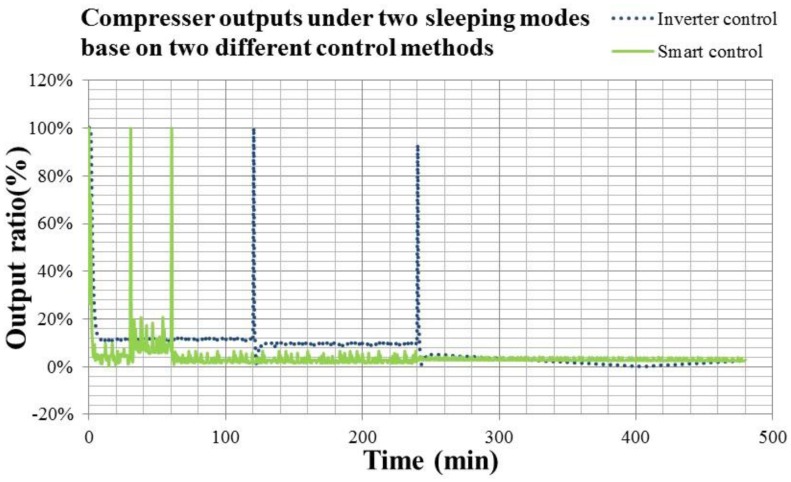
The compressor output of sleeping functions in the night under the Inverter and smart controls.

Figure 21 shows the temperature response of sleeping functions in the night under the Inverter and smart controls. Figure 22 shows the compressor output of sleeping functions in the night under the Inverter and smart controls.

In Figures 21 and 22, the dotted line is the information of Inverter control, and the solid line is that of the smart control. Comparing the results in Figures 21 and 22, it is noted that the smart air conditioner with wearable devices could detect the human temperature and action during sleep for determining the sleeping state and adjusting the sleeping function flexibly. When the human is in a deep sleep, the body temperature would decrease. The air conditioner may increase the temperature setting accordingly for maintain human health. The compressor output of the smart air conditioner in Figure 22 is 46.9% less than that of convertible frequency one. Therefore, the sleeping function optimized by the smart air conditioner with wearable devices is capable of energy conservation and health care.

## 6. Conclusions

From the theoretical analysis and experimental results, the design of a smart air conditioner with mobile phones and wearable devices could be carried out the intention causing control as the a significant improvement of air conditioner technology. There are some conclusions and recommendations for the smart air conditioners as follows:

(1) The total compressor output of a smart air conditioner is 48.4% less than the fixed frequency one. Indoor temperature can be controlled accurately with errors less than 0.1 °C. Rapid cool down can be achieved in 2 min to the optimized indoor capacity after occupants enter.
(2) The intention causing control of smart air conditioner could be practiced by combing with the GPS, personal schedule and setting information of the mobile phone for the optimized setting of compressor output.
(3) The smart air conditioner with wearable devices could detect the human temperature and action during sleep for determining the sleeping state and adjusting the sleeping function flexibly. The sleeping function optimized by the smart air conditioner with wearable devices could reduce the energy consumption up to 46.9% and maintain the human health.

Based on these results, the smart air conditioner with mobile phones and wearable devices could carried out the intention causing control as a significant improvement of air conditioner technology, and be improved for human comfort and energy conservation in the coming future.

## Figures and Tables

**Table 1.** Specification of smart socket used for measuring compressor output.

**Items**	**Unit**	**Value**
Measuring voltage	V	110, 220
Rated current	A	AC 15
Frequency	Hz	50–60
Accuracy	V, A, W, Wh	±1.0%
Communication		Zigbee wireless

**Table 2.** Specification of temperature sensor and anemometer (Testo 435-2).

**Temperature Sensor**		**Anemometer**	
Measuring range	−200 to +400 °C	Measuring range	0 to +60 m/s
Accuracy	±0.3 °C (−60 to +60 °C)	Resolution	0.01 m/s (60 vane)
Resolution	0.1 °C		

## Notes

Author Contributions Chin-Chi Cheng coordinated the experimental work, analyzed the experimental data, and contributed to the writing and organization of the manuscript. Dasheng Lee proposed the research topic and the intension causing control approach, prepared the initial draft of the manuscript and supervised the study.
